# Cost-effectiveness of neuromuscular electrical stimulation for the treatment of mild obstructive sleep apnea: an exploratory analysis

**DOI:** 10.1017/S0266462323000272

**Published:** 2023-06-06

**Authors:** Shan Liu, Khoa N. Cao, Abigail M. Garner, Naresh M. Punjabi, Jan B. Pietzsch

**Affiliations:** 1 Department of Industrial and Systems Engineering, University of Washington, Seattle, WA, USA; 2 Wing Tech Inc., Menlo Park, CA, USA; 3 Division of Pulmonary, Critical Care, and Sleep Medicine, University of Miami, Miller School of Medicine, Miami, FL, USA

**Keywords:** Sleep apnea, obstructive, electrical stimulation therapy, continuous positive airway pressure, cost-benefit analysis, economics

## Abstract

**Objectives:**

To assess the potential cost-effectiveness of neuromuscular electrical stimulation (NMES) for treatment of mild obstructive sleep apnea (OSA).

**Methods:**

A decision-analytic Markov model was developed to estimate health state progression, incremental cost, and quality-adjusted life year (QALY) gain of NMES compared to no treatment, continuous airway pressure (CPAP), or oral appliance (OA) treatment. The base case assumed no cardiovascular (CV) benefit for any of the interventions, while potential CV benefit was considered in scenario analyses. Therapy effectiveness was based on a recent multi-center trial for NMES, and on the TOMADO and MERGE studies for OA and CPAP. Costs, considered from a United States payer perspective, were projected over lifetime for a 48-year-old cohort, 68% of whom were male. An incremental cost-effectiveness ratio (ICER) threshold of USD150,000 per QALY gained was applied.

**Results:**

From a baseline AHI of 10.2 events/hour, NMES, OA and CPAP reduced the AHI to 6.9, 7.0 and 1.4 events/hour respectively. Long-term therapy adherence was estimated at 65-75% for NMES and 55% for both OA and CPAP. Compared to no treatment, NMES added between 0.268 and 0.536 QALYs and between USD7,481 and USD17,445 in cost, resulting in ICERs between USD15,436 and USD57,844 per QALY gained. Depending on long-term adherence assumptions, either NMES or CPAP were found to be the preferred treatment option, with NMES becoming more attractive with younger age and assuming CPAP was not used for the full night in all patients.

**Conclusions:**

NMES might be a cost-effective treatment option for patients with mild OSA.

## Introduction

Obstructive sleep apnea (OSA) is a sleep-related breathing disorder characterized by repetitive complete (apnea) or partial (hypopnea) obstructions of the upper airway, resulting in oxygen desaturation and/or arousals from sleep (1;2). Due to lifestyle, aging, and a rise in obesity, the prevalence of OSA has risen over the past few decades, with a reported prevalence of 33.9 percent in men and 17.4 percent in women aged 30–70 years in 2013 (3). Established risk factors include male sex, older age, obesity, race/ethnicity, and family history (1). Untreated OSA has been associated with several cardiovascular (CV) sequelae, including hypertension, atrial fibrillation, heart failure, coronary artery disease, and CV mortality, in addition to impaired health-related quality of life and daytime sleepiness (1;4).

OSA severity is assessed by the apnea-hypopnea index (AHI), which is the number of apnea and hypopnea episodes per hour during sleep. Mild OSA is defined as an AHI between 5 and <15 episodes per hour. A recent scientific statement from the American Heart Association recommends that all patients, including those with mild OSA, should be considered for treatment (1). Continuous positive airway pressure (CPAP) has long been considered the mainstay of OSA treatment (5). Oral appliances (OA) have provided an effective alternative for mild-to-moderate OSA and patients who cannot tolerate CPAP (5). Both therapies are effective but have been associated with significant long-term adherence challenges, especially in mild OSA (6–8). Further, nightly therapy compliance remains challenging, with patients on treatment often using their devices for only part of the night and thus not achieving the full theoretical treatment benefit (9).

Neuromuscular electrical stimulation (NMES) is emerging as a noninvasive daytime therapy for mild OSA. Recently, daytime stimulation with a novel intraoral neuromuscular stimulation device (eXciteOSA®, Signifier Medical Technologies Ltd, London, UK) has been investigated for the treatment of mild OSA (10;11). NMES treatment with the eXcite OSA device involves noninvasive transoral stimulation for 20 min per day for a period of 6 weeks to increase the endurance of the tongue muscles to prevent the collapse of the tongue during sleep (10;12;13). Repeat treatment at specified follow-up periods is indicated to maintain the desired effect over time. In the studies conducted to date, NMES was found to be associated with improvement in objective and subjective measures of patient and bed partner sleep quality and patient daytime somnolence (10;11;13). Due to the growing evidence that mild OSA is associated with clinical implications, healthcare stakeholders need to understand the clinical benefits and cost-effectiveness of available therapies. The objective of the current study was to conduct an exploratory analysis of the potential cost-effectiveness of NMES compared to CPAP, OA, and no treatment in the American healthcare system.

## Methods

### Overview

A Markov model previously published for evaluation of diagnostic and therapeutic strategies in OSA (14), and subsequently used to explore the cost-effectiveness of novel treatment modalities for moderate-to-severe OSA in the United States and Germany (15;16) was adapted for the current analysis, with the general model structure widely maintained as structure and events also apply to mild OSA. The analysis model tracked the progression of the cohort through relevant health states based on multivariable risk equations, with a lifetime horizon modeled based on the chronic nature of the condition and its treatment and the long-term risk implications of treatment. Event risks were adjusted to reflect those expected in a mild OSA population. The base case analysis assumed treatment was not associated with any CV benefit, whereas a second and exploratory scenario evaluated the implications of potential treatment-associated CV benefit. No institutional review board (IRB) or ethics committee approval was required for the study.

### Model structure

The analysis model comprised five health states and tracked the monthly occurrence of stroke, myocardial infarction (MI), motor vehicle accidents (MVA), and death based on multivariable risk equations, and integrated poststroke and post-MI states to account for different mortality risks and costs. A schematic representation of the computational model is provided in the Supplementary Materials. Table 1 provides a summary of key model inputs. CV event risks in patients with mild OSA were assumed to be elevated compared to a general, non-OSA population. Specifically, a hazard ratio (HR) of 1.23 was applied for cardiac events that was calculated from regression analyses of data reported in a 10-year outcomes study, and an HR of 1.74 based on data from the Sleep Heart Health Study (21;30). As previously noted, the base case analysis did not assume any treatment-related reductions of CV risks, whereas a scenario analysis assumed some reduction of these risks, with each therapy’s effectiveness in reducing these excess risks estimated from the aforementioned regression analysis that linked AHI and CV risk (see supplementary materials and prior publication) (16). Under therapy compliance, the calculated reduction in excess risk was 59.2 percent for NMES (based on an AHI reduction from 10.2 to 6.8), 57.6 percent for OA (based on an estimated reduction in AHI from a common baseline of 10.2 to 7.0 events per hour, derived from effectiveness reported in the TOMADO trial (34), assuming nightly compliance with therapy), and 100 percent for CPAP (based on an estimated reduction in AHI from common baseline of 10.2 to 1.4 per hour, derived from effectiveness reported in the MERGE trial, assuming nightly compliance with therapy) (33). These effectiveness assumptions were further adjusted through multiplication with therapy-specific adherence. The proportion of the cohort assumed to be nonadherent returned to baseline risk levels identical to those incurred by untreated patients, as no subsequent treatment pathways were modeled after stopping one particular treatment.

**Table 1.** Input parameters

	Parameter value	References
*Cohort characteristics*		
Age	48	Nokes et al. (13)
Male gender (percent)	68	
*Risks associated with events and modeled states*		
MVA annual risk, males	0.019 (0.01–0.027)	Chang et al. (17); Blincoe et al. (18)
MVA annual risk, females	0.008 (0.004–0.013)	
Probability of death from an MVA with injury or death	0.0079	
Prevalence of hypertension, males (varies by age, 40–70 years)	0.239–0.633	Cutler et al. (19)
Prevalence of hypertension, females (varies by age, 40–70 years)	0.199–0.788	
Rate (annual per person, until age 85) for incident hypertension, males	0.0195 × ln(Age)–0.0551	Cutler et al. (19)
Rate (annual per person, until age 85) for incident hypertension, females	(2.5 × 10^–7^) × Age^2.948^	
Rate (annual per person, until age 85) for incident MI, males	(2.0 × 10^–12^) × Age^5.236^	Pietzsch et al. (16)
Rate (annual per person, until age 85) for incident MI, females	(9.0 × 10^–17^) × Age^7.423^	
Hazard ratio for incident MI, with hypertension	2.53	Psaty et al. (20)
Probability of 28-day mortality after MI, varies by age	0.075–0.22	Marin et al. (21)
HR for all-cause mortality, life after MI	4.4	Roger et al. (22)
Rate (annual per person, until age 85) for incident stroke	(3.1 × 10^–13^) × Age^5.569^ (±20% rate)	Sanders et al. (23); Anderson et al. (24)
Hazard ratio for incident stroke, with hypertension	2.78 (2.0–4.6)	Psaty et al. (20)
Probability of 28-day mortality after stroke, varies by age	0.0.05–0.22	Joundi et al. (25)
HR for all-cause mortality, life after stroke	2.2	Dennis et al. (26)
HR for MVA causing injury or death	3.0	George et al. (27); Barbé et al. (28)
HR for developing hypertension	1.8	O’Connor et al. (29)
HR for incident MI	1.24	Marin et al. (21)
HR for incident stroke	1.74	Redline et al. (30)

Variable	Base case	References
*NMES effectiveness* (percent returning to non-OSA risk level [1.0 = 100% effectiveness])		
CVD (hypertension, MI, stroke)	0.61	Based on AHI reduction from 10.2 to 6.8 events/hr. from Marin et al. (21)
MVA	1.0	George et al. (27); Tufts (31)
Long-term adherence (percent)	75 at 3 years, then stable	Assumption based on observed adherence in treated population from Bakker et al. (32)
*CPAP effectiveness* (percent returning to non-OSA risk level [1.0 = 100% effectiveness])		
CVD (hypertension, MI, stroke)	0.58	Based on AHI reduction from 10.2 to 7.0 events/hr. Institute for Quality and Efficiency in Health Care (IQWiG) (8); Wimms et al. (33)
MVA	1.0	George et al. (27); Tufts (31)
Long-term adherence (percent)	55 at 3 years, then stable	Assumed to be same as CPAP compliance, based on Jacobsen et al. (7)
*OA effectiveness* (percent returning to non-OSA risk level [1.0 = 100% effectiveness])		
CVD (hypertension, MI, stroke)	1.0	Based on AHI reduction from 10.2 to 1.4 events/hr. Schwab et al. (5); Institute for Quality and Efficiency in Health Care (IQWiG) (8); Quinell et al. (34)
MVA	1.0	George et al. (27); Tufts (31)
Long-term adherence (percent)	55 at 3 years, then stable	Assumed to be the same as CPAP compliance based on Jacobsen et al. (7)
*NMES costs*		
NMES therapy initiation (eXciteOSA setup (controller and mouthpiece), one E&M visit, sleep study)	2,181	Manufacturer-provided suggested retail price of eXciteOSA NMES therapy: $1,500 device and $150 mouthpiece; sleep study, office visit per current fee schedule
NMES controller replacement (every 5 years)	1,631	Manufacturer-provided suggested retail price of eXciteOSA controller $1,500, plus office visit
Annual cost of NMES mouthpieces	600	Manufacturer-provided suggested retail price for mouthpieces, per quarter: $150.00
Cost of annual office visit	131	CPT 99214, one visit per year
*OA costs*		
Therapy initiation (device cost paid by Medicare plus one physician visit Level 4 (CPT 99214), sleep study	1,962	2022 fee schedules
OA device replacement (every 5 years)	1,562	2022 DME fee schedule
Cost of annual office visit	131	CPT 99214, one visit per year
*CPAP costs*		
Therapy initiation (device cost paid by Medicare plus one physician visit Level 4 (CPT 99214), sleep study	1,048	$649.25 (capped rental amount for CPAP device (DME E0601), plus sleep study $399 per current fee schedules
Disposables and accessories, per year	965	Current DME fee schedule, considering 75 percent of allowable amount
Cost of annual office visit	131	CPT 99214, one visit per year
*Event costs*		
Nonfatal MVA with injuries	9,106	Blincoe et al. (18)
Fatal MVA	34,116	
Acute MI	17,299	Krumholz et al. (35)
Acute stroke	25,748	Johnson et al. (36); Salata et al. (37)
Baseline healthcare cost, annual	2,984–19,684	Meara et al. (38)
Well	0	
Hypertension	891	Davis et al. (39)
Post-MI	6,158	Ito et al. (40)
Poststroke, post year 1	31,371	Johnson et al. (36)
*Utilities*		
Age adjustment factor, decreases by age (40–80 years) and is applied to all utilities listed below	0.871–0.736	Sullivan et al. (41)
Untreated mild OSA	0.86	Derived from Wimms et al. (33)
Treated mild OSA (NMES; OA; CPAP)	0.89; 0.88; 0.89	See Supplementary Materials for detailed calculations
MI (first year, subsequent)	0.76, 0.88	Glasziou et al. (42); Aasa et al. (43)
Stroke (first year, subsequent)	0.63, 0.63	Grosso et al. (44); Darlington et al. (45)

### Clinical data

Cohort characteristics were derived from the mild OSA subcohort (*N* = 65) of a recent multicenter study of participants treated with NMES (13). In this cohort (mean age 48 years, 68 percent male gender), the mean baseline AHI of 10.2 events per hour was reduced to 6.8 events per hour (13). For the “no treatment” strategy, patients were assumed to remain at the baseline AHI of 10.2. To identify effectiveness assumptions for the CPAP and OA comparator strategies, the findings of a recent systematic search were used (8). Limiting the studies identified in that search to those with a mean baseline AHI of 15 events per hour or less yielded one study each for CPAP and OA––the MERGE trial and the TOMADO study (33;34). MERGE was a multicenter, parallel, randomized controlled trial that enrolled patients from 11 UK sleep centers with an AHI of 5–15 events per hour which closely resembled the cohort characteristics of the NMES study. In MERGE, 115 patients were allocated to CPAP and *N* = 118 to standard care (33). At a mean follow-up of 3 months, the CPAP group reduced their AHI from baseline of 10.6 to 1.5 events per hour while on therapy. The TOMADO study was a crossover randomized controlled trial studying OA use in 83 patients with OSA (34). The mean pretreatment AHI of 13.8 events per hour was reduced to 9.5 events per hour. Cohort characteristics of the MERGE and TOMADO studies were closely comparable to the NMES cohort (see Supplementary Materials). The long-term therapy adherence for CPAP was assumed to be 55 percent, as reported at 3 years for the mild OSA subcohort in a retrospective study of *N* = 695 newly diagnosed OSA patients (7). Based on a study that demonstrated comparable adherence between OA and CPAP over time, OA was assumed to have the same long-term adherence as CPAP. For NMES, a recent study reported that the study population completed a therapy session on 83 percent of available days, over an average follow-up period of 6 weeks (13;32). Building on this information and the fact that NMES is used during the day, the analysis explored a base case scenario of 75 percent long-term adherence for NMES, and a low adherence (LA) scenario of 65 percent.

In the scenario analysis that explored the potential effects of cardiovascular benefit, CPAP was assumed to reduce OSA-associated cardiovascular risk completely in the base case. Additional computations were performed for assumed lower cardiovascular risk reduction benefits of only 80 and 60 percent that might be expected if part of the CPAP-adherent patients did not use their device for the full night, and a scenario that considered only 50 percent of the CV benefit calculated for the respective therapy.

### Health-related quality of life

Estimates for health-state-specific utility values were based on data from the published literature. Values ranged from 0.63 (poststroke) to 1.0 (asymptomatic patient with no CV morbidity and a nonelevated AHI before age adjustment) (see Table 1). Utility estimates for untreated, mild OSA were derived from the MERGE study, which collected baseline EQ-5D values for patients with mild OSA (33). Study-reported improvements in the Epworth Sleepiness Scale (ESS) were used to calculate EQ-5D improvements based on a previously published algorithm (46). See Supplementary materials.

### Resource use and costs

The NMES treatment cost, whilst not yet fully established in the American healthcare system, was based on assumptions about therapy cost (controller and mouthpiece) and, where applicable, existing reimbursement rates (e.g., home sleep test conducted prior to therapy initiation). Importantly, in the absence of allowable reimbursement amounts, NMES device costs were informed by manufacturer-suggested retail prices. OA and CPAP treatment costs––conversely––were based on current Medicare fee schedules as a proxy for true cost. For CPAP accessories/disposables, 75 percent of the maximum allowable monthly cost was used in the base case based on expert interviews. For NMES, OA, and CPAP strategies, a device lifetime of 5 years was considered, after which the devices would be replaced at the expense of Medicare. See Table 1 for detailed assumptions.

The model accounted for both acute event costs (MI, stroke, and MVA) and health-state-specific costs. All costs were derived from published literature and publicly available data (see Table 1). The consumer price index inflator was used to adjust historical costs to 2021 costs where applicable. Both costs and outcomes were discounted at 3.0 percent per annum, in line with recommendations for health-economic analysis (23).

### Model outcomes and willingness-to-pay threshold

The primary analysis outcomes were strategy-specific total costs, quality-adjusted life year (QALY) gains, and the resulting incremental cost-effectiveness ratio (ICER), measured in dollars per QALY gained over the patient’s lifetime. In line with recommendations by the joint statement of ACC/AHA on cost-effectiveness, an intervention with an ICER of up to USD150,000 per QALY gained was considered “of value” (47). Consequently, a willingness-to-pay threshold of USD150,000 per QALY gained was applied to evaluate cost-effectiveness.

### Scenario, sensitivity, and threshold analyses

In addition to the base case analysis and the exploratory scenario that considered CV benefit, several other scenarios were calculated. These included calculations for a hypothetical cohort aged 65 years to more closely resemble a Medicare population. To evaluate the effect of variation in therapy adherence, CPAP/OA adherence and NMES adherence were varied between 100 and 45 percent, and 100 and 55 percent, respectively, in two-way sensitivity analyses. These were performed to determine which of the strategies were preferred for different combinations of therapy adherence assumptions, with preference determined based on a willingness-to-pay threshold of USD150,000 per QALY gained. Where this two-way analysis considered CV benefit, three assumptions of CPAP-associated CV risk reduction were explored to reflect partial compliance in CPAP-adherent patients. These scenarios included a 60, 80, and 100 percent effectiveness assumption resulting from only partial therapy use during the night. Further, in addition to the 75 percent Medicare allowable amount for CPAP accessories/disposables, 50 and 100 percent were explored as scenarios.

A range of one-way sensitivity analyses were conducted to evaluate the effect of parameter uncertainty on analysis results. A sensitivity analysis was calculated to determine cost assumptions where NMES might become cost-saving versus CPAP therapy, on the grounds that NMES costs assumed in the current analysis may be higher than negotiated Medicare rates.

## Results

**Figure 1.**
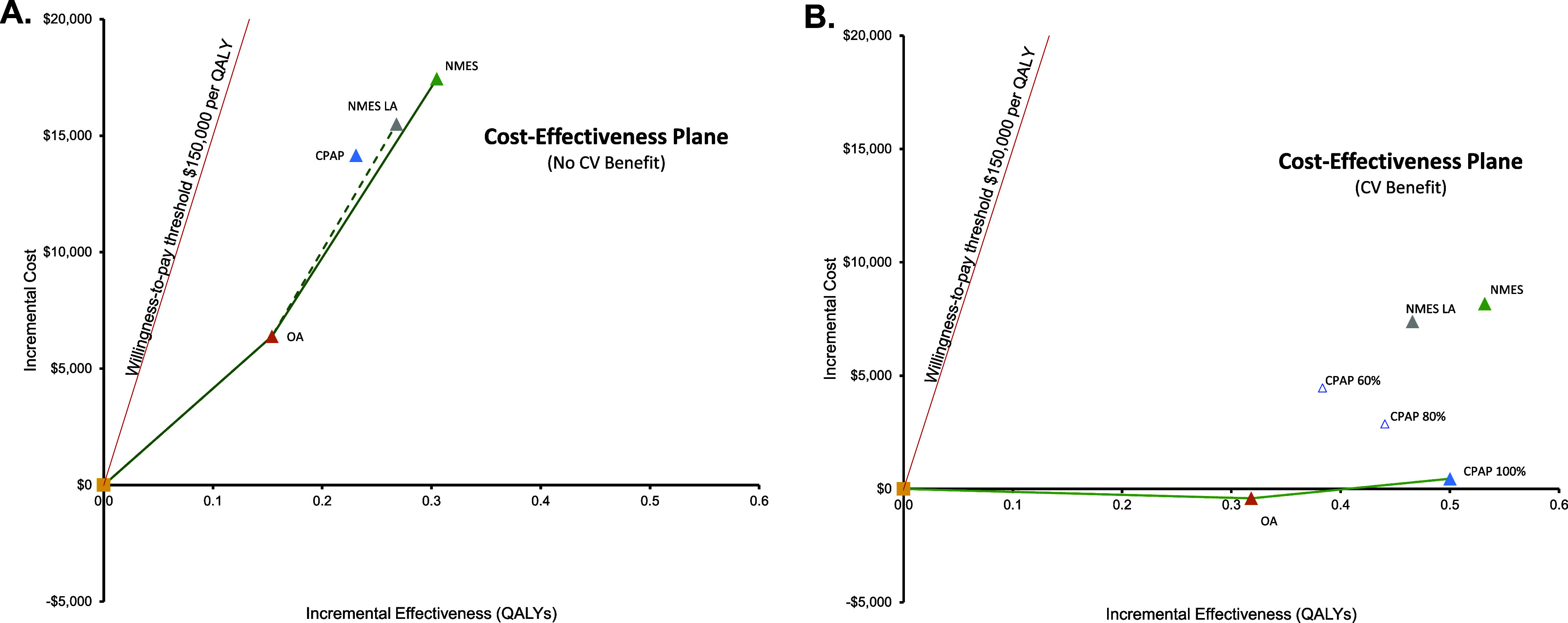
Lifetime cost-effectiveness estimates for NMES, OA, and CPAP versus no treatment, base case (A) and exploratory analysis with consideration of potential therapy-associated cardiovascular benefit (B). CPAP: continuous positive airway pressure; NMES: neuromuscular electrical stimulation; OA: oral appliance; LA: low adherence assumption for NMES, 65 percent long-term; CPAP 100/80/60 percent: Cardiovascular risk reduction with CPAP 100/ 80/60 percent, respectively. Interpretation of figures: On the *x*-axis, incremental QALYs of the respective intervention versus no treatment are shown, on the *y*-axis incremental lifetime costs of each therapy compared to No treatment. The green lines denote the “efficient frontier” that includes all interventions not dominated by the others. In Figure A, NMES is the preferred option, as it is cost-effective versus the next-best option in both the standard and low-adherence scenario. In Figure B, NMES is not cost-effective relative to CPAP, if CPAP nightly use is 100 percent, However, NMES would be cost-effective if CPAP nightly use was only 80 or 60 percent.

**Figure 2.**
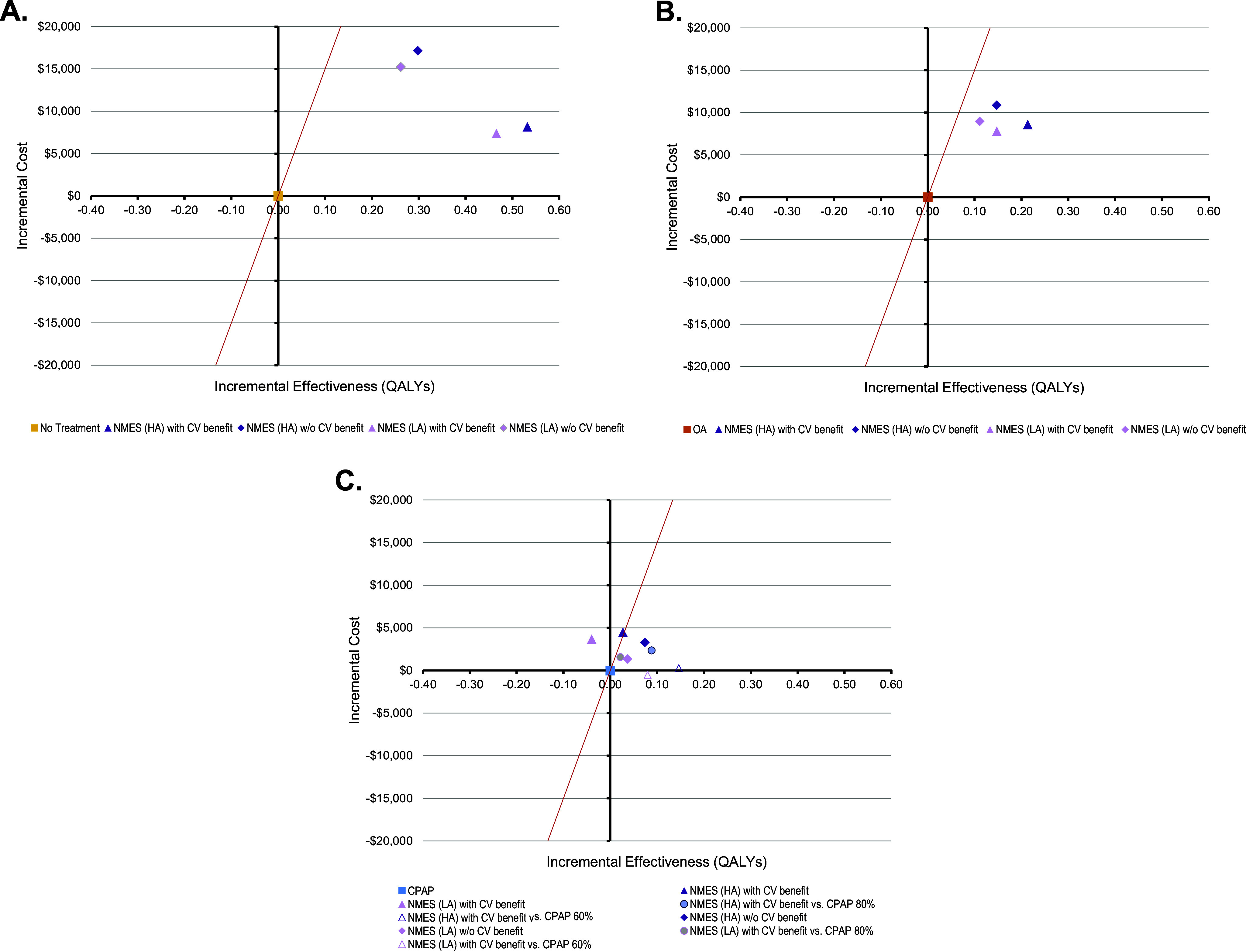
Lifetime cost-effectiveness estimates for NMES versus no treatment (A), NMES versus OA (B), and NMES versus CPAP (C). CPAP: continuous positive airway pressure; NMES: neuromuscular electrical stimulation; OA: oral appliance; LA: low adherence assumption for NMES, 65percent long-term; CPAP 100/80/60 percent: Cardiovascular risk reduction with CPAP 100/ 80/60 percent, respectively. Interpretation of figures: On the *x*-axis, incremental QALYs of the respective intervention versus no treatment are shown, on the *y*-axis incremental lifetime costs of each therapy compared to no treatment. The red line denotes the willingness-to-pay (WTP) threshold of USD150,000 per QALY. Scenarios are cost-effective versus no treatment as long as they lie to the right of the WTP line.

Compared to no treatment, NMES was the preferred treatment option, adding 0.30 QALYs (13.57 versus 13.26) and USD17,445 in costs (USD527,853 versus USD510,407) over the patients’ lifetime, resulting in an ICER of USD57,219 per QALY gained (Table 2). For the NMES low adherence scenario, the ICER was minimally higher at USD57,844 per QALY gained, based on 0.27 (13.53 versus 13.26) QALYs gained and incremental costs of USD15,498 (USD525,905 versus USD510,407), rendering NMES a cost-effective intervention. Relative to CPAP, NMES added 0.07 QALYs (low adherence scenario 0.03 QALYs) at an added cost of USD3,291 (USD1,324), leading to a cost-effective ICER of USD44,528 (USD36,160) per QALY (Figures 1 and 2). For the older treatment age of 65 years, NMES versus no treatment resulted in an ICER of USD71,312 (low adherence scenario USD72,198), NMES versus OA in an ICER of USD73,301 (USD79,990), and NMES versus CPAP in an ICER of USD66,335 (USD67,973) per QALY gained. See Table 2 for full results reporting of scenario analyses.

**Table 2.** Base case and scenario analysis results

	Lifetime cost (USD) and QALYs				Resulting ICERs		
	No treatment	NMES	OA	CPAP	NMES versus No treatment	NMES versus OA	NMES versus CPAP
Base case (age 48 years)	510,407	527,853	516,792	524,562	57,219	73,301	44,528
	13.26	13.57	13.42	13.49			
Base case undiscounted	933,825	960,565	943,040	955,975	61,035	79,904	41,748
	20.70	21.14	20.92	21.03			
10-year analysis horizon	144,420	152,517	147,781	150,934	49,590	60,728	44,799
	6.02	6.19	6.11	6.15			
Age 65 years	483,012	495,323	487,829	492,753	71,312	66,076	66,335
	8.31	8.48	8.37	8.44			
Low NMES long-term adherence (65%)	510,407	525,886	516,792	524,562	57,842	80,045	36,160
	13.26	13.53	13.42	13.49			
CPAP supplies cost at 75 percent of Medicare-allowable ($966 per yr.)	510,407	527,853	516,792	520,905	57,219	73,301	94,009
	13.26	13.57	13.42	13.49			
NMES cost at 75 percent of MSRP (control unit $1,238, mouthpieces $450/yr.)	510,407	524,203	516,792	524,570	45,249	49,116	NMES dominant
	13.26	13.57	13.42	13.49			
CPAP supplies cost at 100 percent of Medicare-allowable ($1,288 per yr.)	510,407	527,853	516,792	528,230	57,219	73,301	NMES dominant
	13.26	13.57	13.42	13.49			
Scenario with CV benefit considered (CPAP nightly compliance 100%)	510,407	518,683	509,981	514,213	15,436	40,078	164,581
	13.26	13.80	13.58	13.77			
Scenario with CV benefit considered (CPAP nightly compliance 60%)	510,407	518,683	509,981	518,427	15,436	40,078	1,747
	13.26	13.80	13.58	13.65			
Scenario with 50 percent of CV benefit considered (CPAP nightly compliance 100%)	510,407	523,321	513,425	519,467	30,994	12,902	70,064
	13.26	13.68	13.50	13.62			
NMES cost at 75 percent of MSRP (control unit $1,125, mouthpieces $450/yr.)	510,407	524,203	516,792	524,562	45,249	49,116	NMES dominant
	13.26	13.57	13.42	13.49			
NMES cost at 50 percent of MSRP (control unit $750, mouthpieces $300/yr.)	510,407	520,554	516,792	524,562	33,279	24,931	NMES dominant
	13.26	13.57	13.42	13.49

Where potential CV benefit was considered, NMES-associated QALY gains versus no treatment were larger (0.54 and 0.47 QALYs for base case and NMES low adherence scenario), and NMES cost-effectiveness versus no treatment was more favorable (Figures 1B and 2A). Compared to CPAP, incremental QALY gains with NMES were either positive or negative, depending on CPAP CV risk reduction effectiveness, resulting in a range of scenarios that rendered NMES either cost-effective or not cost-effective versus CPAP (Figure 2C). Compared to OA, NMES was found cost-effective in both the base case and the CV benefit scenario (Figure 2B).

**Figure 3.**
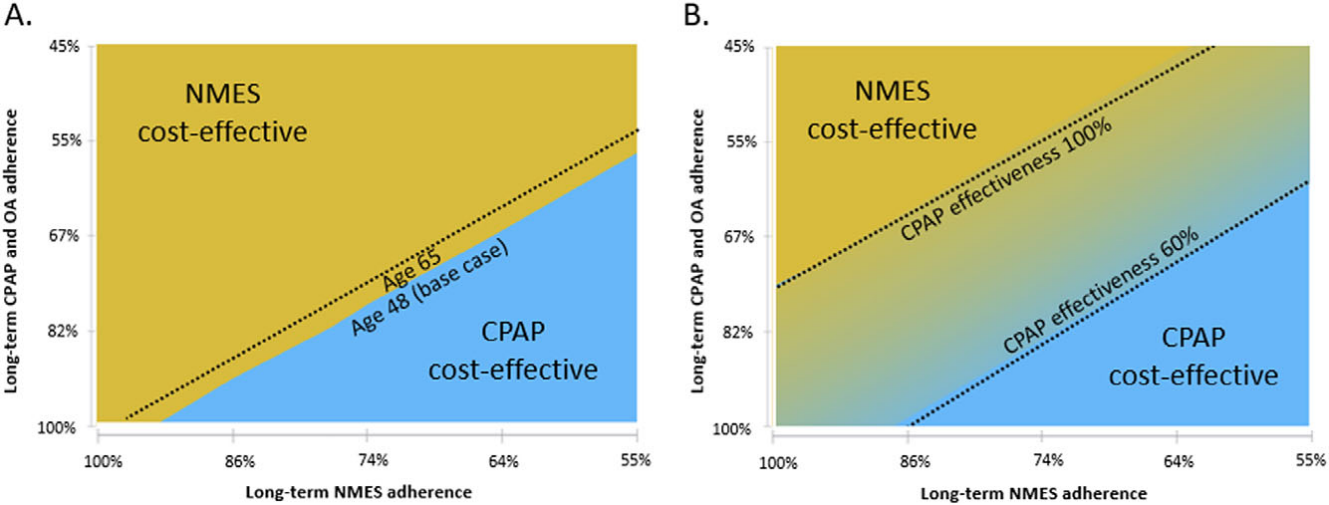
Two-way sensitivity analysis of the effect of variation in long-term CPAP/OA adherence versus NMES adherence on cost-effectiveness. Male cohort, no cardiovascular benefit scenario (A) and male cohort, cardiovascular benefit scenario (B). CPAP: continuous positive airway pressure; NMES: neuromuscular electrical stimulation; OA: oral appliance. Interpretation: The graphs show which therapy is preferred (at considered willingness-to-pay threshold of USD150,000 per QALY gained) for various combinations of long-term therapy adherence for NMES (*x*-axis) and CPAP and OA (*y*-axis, varied concurrently for both CPAP and OA).

For the adherence scenarios explored in two-way sensitivity analyses, only NMES or CPAP were the preferred strategies for all explored combinations of therapy adherence. An older cohort age of 65 marginally increased the favorability of CPAP (Figure 3A). At the same time, reduced CPAP CV effectiveness expected from only partial use of CPAP therapy during the night increased the favorability of NMES, when CV benefit was considered (Figure 3B).

## Discussion

In this simulation, a comprehensive analysis framework for the evaluation of therapy options for mild OSA was presented. NMES, as a novel treatment approach not relying on nightly therapy use––and thus meeting an unmet clinical need––was found to be cost-effective versus no treatment and or the use of OA. Compared to CPAP, NMES provided improved outcomes in most analyses, and was found cost-effective versus CPAP in several but not all scenarios. Long-term adherence assumptions, CPAP therapy compliance during the night and cost assumptions were identified as key drivers determining the cost-effectiveness of NMES versus CPAP. Importantly, in the absence of negotiated allowable amounts, the current analysis relied on present manufacturer-suggested retail prices for the NMES device and disposables. A sensitivity analysis demonstrated cost savings versus CPAP when NMES reimbursement was at or below 75 percent of the base case assumption. Actual long-term prices might be lower. Thus, the current analysis findings are likely very conservative and might therefore underestimate the true long-term cost-effectiveness of NMES.

Only three prior studies have investigated the cost-effectiveness of treatments for mild OSA (46;48;49). A study from France assessed the cost-effectiveness of treatments for mild-to-moderate OSA. In that study, analyses were conducted separately for a low CV risk cohort and for a high CV risk cohort that was 56 years of age. The authors of that study reported a QALY gain of CPAP versus no treatment of 0.187 in mild OSA patients at low CV risk, closely in line with the QALY gains of 0.134 and 0.231 projected in this study for cohorts 48 and 65 years of age (48). McDaid et al. (46) in a UK health technology assessment conducted in the early 2000s, evaluated the cost-effectiveness of CPAP versus no treatment. In a scenario analysis of mild OSA patients 50 years of age, their study (“the York model”) found a lifetime gain of 0.13 QALYs, somewhat lower than the 0.20 QALYs projected in the current study for the slightly younger cohort aged 48 years. Finally, Sharples et al, in an economic analysis of the TOMADO trial that also included a model-based assessment of CPAP, projected a QALY gain of 0.304 for CPAP versus no treatment in mild-to-moderate OSA, and again found very similar QALYs for OA and CPAP, with an incremental gain of 0.02 QALYs in favor of CPAP (49).

As is well known and confirmed herein, therapy adherence is a key driver of the cost-effectiveness of OSA treatments. This applies particularly in less severe OSA, as evidenced by the study by Jacobsen et al. that found almost half of mild OSA patients had stopped using CPAP within 3 years (7). An equal challenge appears to apply for OA, as evidenced by a review study that pooled data from 21 studies, and found OA compliance between 56 and 68 percent at an average follow-up of 33 months (50). Importantly, even in adherent patients, insufficient nightly therapy compliance might reduce therapy effectiveness, as has been widely discussed and triggered the development of metrics such as the “effective AHI” (51). The current analysis explored these scenarios through consideration of CPAP-associated CV risk reduction effectiveness of only 60 or 80 percent, as opposed to 100 percent assumed in the base case.

NMES treatment with the studied stimulation device eliminates the need for therapy during sleep time, and thus overcomes the challenge of nightly therapy compliance. Although patients need to be compliant with device use during the day for defined periods of time, there are extensive periods where patients achieve clinical benefit without any device interaction or compliance, suggesting that sustained outcomes might be achieved at a lower adherence burden compared to CPAP or OA. Additional data about the multiyear adherence and the resulting sustainable outcomes of NMES in real-world practice will be helpful to confirm the current study’s assumptions. However, it is likely that this characteristic of NMES treatment leads to additional patient benefit that is not fully captured in the current analysis.

The current study builds on a body of evidence about the implications of treated versus untreated mild OSA that is still emerging, with evidence and insight more limited compared to moderate-to-severe OSA. There is increasing recognition that mild OSA should be treated to improve quality of life and, in some patients, particularly those with CV comorbidities, might also play a role in reducing overall CV risks. The recent MERGE trial reported that three months of treatment with CPAP for mild OSA led to statistically significant improvements in the SF-36 Vitality metric by 10 points (95 percent CI 7.2–12.8) (33). These findings extended in subset analysis to very mild OSA patients with improvements in vitality and sleepiness metrics (52). At the same time, the relation between mild OSA and CV events remains controversial. Although a meta-analysis of clinical trials found no significant association between CPAP utilization and some CV events (53), other meta-analyses have found a modest, but consistent blood pressure reduction of 2 mmHg from the utilization of CPAP (54), which would be anticipated to translate to a reasonable reduction in CV events (55). Provided the uncertainties surrounding the CV benefit of treating mild OSA, the base case analysis in this study considered no treatment-associated CV benefit.

This study has several limitations. First, although an emerging body of clinical evidence is available for all three studied interventions, data are still limited in the mild OSA indication. Further, evidence on potential cardiovascular risk reduction in mild OSA is uncertain. The study is thus exploratory in nature rather than definitive. Comprehensive scenario and sensitivity analyses were conducted to provide insight of the effect or remaining parameter uncertainty. Second, where available, Medicare reimbursement was used as a proxy for true cost. Private payer reimbursement might differ, which to a certain extent might affect the cost-effectiveness results. However, such difference in private payer cost might be expected to apply to all therapies. Third, as outlined earlier, current cost assumptions of NMES might be significantly higher than what Medicare will ultimately pay for this intervention once allowable amounts have been set. The current analysis might therefore underestimate the long-term cost-effectiveness of NMES. Fourth, this analysis was exploratory in nature. Future, more definitive studies will benefit from more granular modeling of uncertainty and presentation of probabilistic sensitivity analyses. These were beyond the scope of the current paper. Finally, the analysis considered no sequential scenarios of treatment, for example, a patient who has failed CPAP therapy then advancing to OA or NMES as a subsequent treatment. However, insights from the current analysis might help to at least theoretically estimate the health-economic value proposition of such sequential treatments.

In conclusion, NMES––a noninvasive daytime treatment whose clinical effect is independent of night-time usage––might be a cost-effective treatment option for patients with mild OSA and may be preferred over CPAP or OA depending on adherence and cost assumptions.

## Supplementary material

For supplementary material accompanying this paper visit https://doi.org/10.1017/S0266462323000272. click here to view supplementary material
