# Supplementary material for: Cost-effectiveness of neuromuscular electrical stimulation for the treatment of mild obstructive sleep apnea: an exploratory analysis
Source: Int J Technol Assess Health Care. 2023 Jun 6;39(1):e32. doi: 10.1017/S0266462323000272 (PMC11574537; doi:10.1017/S0266462323000272)
Supplement: Supplementary file 1 [file S0266462323000272sup001.doc]

###### Supplemental Materials/e-Appendix

**Cost-Effectiveness of Neuromuscular Electrical Stimulation for the Treatment of Mild Obstructive Sleep Apnea: An Exploratory Analysis**

**S.1 Cohort characteristics of underlying clinical studies**

###### The manuscript analysis relies on data from Nokes et al, 2022 (NMES), MERGE - Wimms et al.2019 (CPAP), and TOMADO (Quinell et al. 2014).

###### The cohort characteristics in these studies were widely comparable, as seen below:

|  | NMES  (Nokes et al. 2022) | CPAP  (MERGE; Wimms et al, 2019) | OA  (TOMADO; Quinn et al. 2014) |
| --- | --- | --- | --- |
| Age | 48 | 50 | 51 |
| % male | 68 | 70 | 80 |

###### .

**e-Table 1:** Comparison of cohort characteristics of the underlying clinical studies.

**S.2 Model schematic**


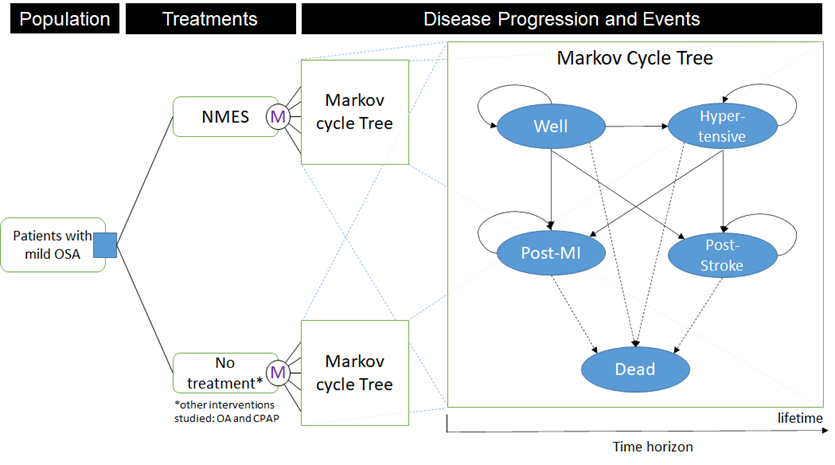


**e-Figure 1**: Schematic depiction (simplified) of the model structure, a combination of decision tree and Markov disease progression model. The same structure applies for modelling of OA and CPAP as additional comparators.

Legend: CPAP: Continuous positive airway pressure; MI myocardial infarction; NMES: Neuromuscular electrical stimulation; OA: Oral appliance; OSA obstructive sleep apnea.

**S.2 Estimation of cardiovascular risk and of therapy effectiveness in reducing these risks**

###### The manuscript analysis, in its base case, does not assume any therapy-associated cardiovascular benefit. However, in scenario analysis, an exploratory analysis was performed that considered cardiovascular benefit. The therapy-specific reduction of the calculated excess cardiovascular event risk was informed by the degree of AHI reduction achieved with each intervention.

###### Following, details on the calculation of the assumed excess event risk are provided, and subsequently assumptions calculated for each therapy’s effectiveness in reducing these excess event risks.

###### Excess MI risk:

###### Marin et al. [^1^](#_ENREF_1) reported long-term cardiovascular outcomes in CPAP-treated vs. untreated male OSA patients. The Marin study included 264 healthy men, 377 simple snorers, 403 with untreated mild-moderate obstructive sleep apnea-hypopnea, 235 with untreated severe disease, and 372 with the disease and treated with CPAP. Subjects were followed for 10 years to compare incidence of fatal and non-fatal cardiovascular events between cohorts. Table A.1 below shows the subset of nonfatal and fatal cardiovascular event rates reported by Marin et al. that are relevant for the current study, and the resulting total event rate, which was computed based on the Marin data.

###### e-Table 2: Overview of subset of data used from Marin et al. study[^1^](#_ENREF_1), and resulting total nonfatal or fatal cardiovascular events per 100 person years.

###### The lower range of AHI derived from the study data were used to estimate a relationship between AHI and cardiovascular event rates, using a non-linear (polynomial) regression function. E-Figure 2 shows this resulting regression function, as well as the Marin et al.-derived cardiovascular event rates.

CV events per 100 person years


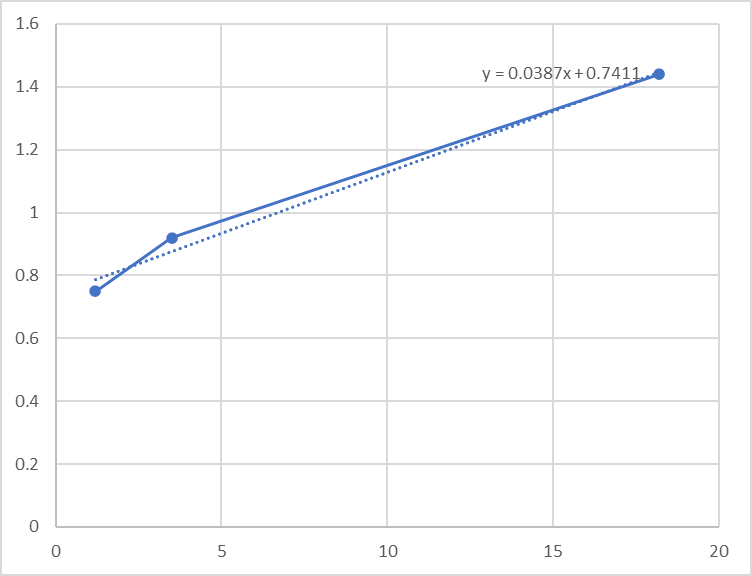


AHI

###### e-Figure 2: Regression analysis-based approximation estimating functional relationship between AHI and cardiovascular event risk. Large dots show data reported in Marin et al.[^1^](#_ENREF_1).

###### Using this functional relationship, an excess risk HR of 1.23 was calculated for the mild OSA population studied in the current analysis (CV event rate 1.14 for AHI of 10.2 events per hour – the baseline of the current study – vs. CV event rate 0.92 for AHI of 3.5, which was assumed to be representative of the general population AHI level).

###### Excess stroke risk:

###### Data from the Sleep-Heart-Health Study[^2^](#_ENREF_2) reported the following hazard ratios (HR) for stroke: HR for AHI 4.1 to 9.4 vs. 0 to 4.1: 1.86 for men, 1.34 for women. HR for AHI 9.5 to 19.0 vs. 0 to 4.1: 1.86 for men, 1.20 for women. Based on the assumed gender mix, a resulting excess risk HR of 1.74 was assumed for calculation purposes.

###### Calculation of each therapy’s effectiveness in reducing these excess cardiovascular risks:

###### For each therapy, AHI reductions from baseline AHI of 10.2 events per hour were calculated as follows:

###### NMES: AHI reduction from 10.2 to 6.8 events per hour (per study data)[^3^](#_ENREF_3), corresponds to an excess risk reduction from HR 1.23 to HR 1.09 relative to general population risk, a reduction of 61% of the excess cardiovascular event risk.

###### OA: Based on the TOMADO study[^4^](#_ENREF_4), the only identified study with a mean baseline AHI of 15 events or less and thus qualifying as a mild OSA study, the ratio of on-treatment AHI vs. baseline AHI was 0.69. Applying this to the modeled baseline of 10.2 events from the NMES study yielded a reduction of AHI to 7.0 on-treatment. Applying a corresponding calculation as outlined for NMES, this results in a reduction of 58% of the excess cardiovascular event risk.

###### CPAP: Based on the MERGE study[^5^](#_ENREF_5), in which AHI was reduced from 10.6 events per hour at baseline to 1.5 events per hour on treatment (or, applied to baseline of 10.2 events per hour, a reduction to 1.4 events per hour. On this basis, a reduction of 100% of the excess cardiovascular event risk was assumed to be achieved with CPAP therapy (in a population therapy-adherent per the MERGE trial).

**S.3 Derivation of therapy-specific utilities**

The therapy-specific utilities shown in Table 1 were derived as follows:

The utility for untreated OSA was derived from the EQ-5D score reported at baseline (AHI of 10.6 events per hour) in the MERGE study: 0.75

After age-adjustment as used in the analysis model, this yields a non-age adjusted utility of 0.86 at baseline.

Subsequently, a change in utility of 0.01 for every point change in the Epworth Sleepiness Scale (ESS) was assumed based on a functional relationship established by McDaid et al. (2009):

OSA untreated (baseline) = Baseline ESS * (-0.01) + 0.89

For NMES, per source data, (“Mean ESS for the whole group reduced from 8.7 to 5.3 (p<0.01, SD 4.1, 95% CI 2.4-4.4)"), a reduction in ESS of 3.4 was observed. This resulted ina utility improvement of 0.03 per the McDaid equation.

For CPAP, ESS in MERGE (Wimms et al, 2019) improved by 3.0, resulting in a utility improvement from baseline of 0.03.

For OA, the following reductions from baseline ESS of 10.1 were observed in the different subgroups of TOMADO (Quinell, 2014): 1.51 (N=81), 2.15 (N=77), 2.37 (N=72). The weighted average reduction in ESS was thus 1.99, resulting in a calculated utility improvement from baseline of 0.02.

As a result, the following utilities were identified:

- No treatment 0.75 (and 0.86 as non-age adjusted estimate)
- NMES 0.78 (0.89)
- CPAP 0.77 (0.89)
- OA 0.77 (0.88)

Note this directly maps to the MERGE study-observed improvement with CPAP of 0.03, confirming validity of the McDaid-based calculations.

###### References:

1. Marin JM, Carrizo SJ, Vicente E, Agusti AG. Long-term cardiovascular outcomes in men with obstructive sleep apnoea-hypopnoea with or without treatment with continuous positive airway pressure: an observational study. *Lancet.* 2005;365(9464):1046-1053.

2. Redline S, Yenokyan G, Gottlieb DJ, et al. Obstructive sleep apnea-hypopnea and incident stroke: the sleep heart health study. *American journal of respiratory and critical care medicine.* 2010;182(2):269-277.

3. Baptista PM, Martínez Ruiz de Apodaca P, Carrasco M, et al. Daytime Neuromuscular Electrical Therapy of Tongue Muscles in Improving Snoring in Individuals with Primary Snoring and Mild Obstructive Sleep Apnea. *Journal of clinical medicine.* 2021;10(9):1883.

4. Quinnell TG, Bennett M, Jordan J, et al. A crossover randomised controlled trial of oral mandibular advancement devices for obstructive sleep apnoea-hypopnoea (TOMADO). *Thorax.* 2014;69(10):938-945.

5. Wimms AJ, Kelly JL, Turnbull CD, et al. Continuous positive airway pressure versus standard care for the treatment of people with mild obstructive sleep apnoea (MERGE): a multicentre, randomised controlled trial. *Lancet Respir Med.* 2020;8(4):349-358.
